# Lectures on the Physical Phenomena of Living Beings

**Published:** 1849

**Authors:** 


### Part 2.—Reviews and Notices of New Works

ARTICLE I.

*Lectures on the Physical Phenomena of Living Beings.* By CARLO MATTEUCCI, Professor in the University of Pisa. with numerous wood-cuts. Translated under the superintendence of JONATHAN PEREIRA M. D. F. R. S. &c. Phiadelphia: Lea and Blanchard. 1848. pp. 388 12 mo. (From the publishers,)

No science has more reason to be proud of her votaries than medicine. Physicians have been behind no other class of philosophers in the investigation of truth; extending their researches in every direction, they have neglected no science that might throw light on the nature and treatment of disease. The world is mainly indebted to physicians for the great discoveries in organic and inorganic chemistry, discoveries which have had not only an important bearing in the healing art, but on many other arts that minister to the comfort and convenience of mankind. If natural philosophy boasts of her Newton, La Place and Herschel; medicine may point with pride to Pare, Galvani, Harvey, Jenner, Berzelius, Liebig and a host of others whose names will be meutioned with respect until gratitude ceases to be a trait of human nature. To the honor of the profession be it said that its members generally are at all times ready to discuss and appreciate the merits of any invention or discovery, and give it at once a practical application to the relief of suffering. No sooner had Galvani demonstrated the influence of electricity organized tissues than physicians in all civilized countries imagined the possibility of making the discovery useful in the treatment of disease, and although they ran into divers absurdities, although keen sighted charlatans made the discovery of the illustrious Italian the means of practising monstrous impositions on the multitude, chaos was soon reduced to order, and the therapeutical value of electricity to a considerable extent defined and established. So it has been with all discoveries relating to our profession. Upon the announcement of any new theory or discovery, experiments have been made with wonderful ingenuity and industry, for the purpose of testing the truth and applicability of the new doctrine.

The work before us has for its object the application of the recent discoveries in chemistry and the general laws of natural philosophy to the explanation of the phenomena of living beings. The high character of Dr. Pereira is a sufficient guaranty that the work is faithfully translated, consequently we may rely with confidence on the work as a correct exposition of the views of the Pisa professor. The work is in many respects new, or perhaps it would be more proper to say that so far as we know, the design is purely original. Many of the items composing the work are contained in various treatises on chemistry and Physiology; these have been collected, properly arranged, and made subservient to the design of the learned author.

It has been for ages a problem with physiologists and scientific men, to solve this question; what is life? What is called the vital principle has in vain been sought in the brain and its appendages, and in the circulating system. Various have been the theories which have been from time to time advanced, supported with much ingenuity, and successfully demolished. Observing that organization was necessary to support life, Richerand supported with much ability the no less unphilosophical than atheistical dogma that life was the result of organization; on the other hand, the fact that life was essential to the perpetuity of organization, led others to the equally fallacious conclusion that organization was the result of life. Others again, observing the wonderful effects of electricity on the animal economy, were induced to believe that the long sought agent was to be found in the electric fluid—that the brain was the generator, and the nerves the conductors which distributed the fluid—that all diseases were but disturbances of the electric equilibrium, and that a man was ailing in proportion as he was positive or negative. Prof. Matteucci avoids all discussion on this point, confining himself to a detail of numerous and varied experiments both on the dead and living animal, giving a few brief general conclusions as he goes along, without involving himself in the defence of any particular doctrines or opinions.

He sets out by observing that living beings are endowed with the general properties of all natural bodies; that they are extended, impenetrable, divisible, and porous. This is true enough; but living beings have properties peculiar to themselves; properties which, in a remarkable manner, distinguish them from inorganic bodies, and which should be taken into the account by the experimenter. Inorganic bodies are homogeneous in their character; organized bodies are heterogeneous. One who sees a cubic foot of granite, sees all that composes the rock from which it was quarried, but he would know very little of the structure of an animal by seeing only a portion of it. The chemical character of organic and inorganic bodies is essentially different. The phenomena of organized bodies, when under the influence of the organic or vital force, are essentially distinct from the purely chemical processes which are set up after death, and result in the destruction of the organization; hence laws deduced from experiments on dead animal tissues, are not to be regarded as universal laws in the explanation of vital phenomena; and it would be difficult if not impossible to state with precision how far such conclusions are really applicable to the living animal economy. We do not assume that because of these differences, caloric, electricity, light, and chemical affinity act entirely differently in the two classes of bodies; nevertheless we suggest that the vital force in many instances modifies to a greater or less extent, the effects of the above named agents. Again—nearly all the experiments instituted with the purpose of elucidating the laws of physiology and organic chemistry, have been peformed upon the lower animals. Now, without stopping to discuss the humanity of subjecting brutes to torture, we will observe that the results of such experiments are to be received with caution, and we are bound to doubt whether the laws of human animal organization are identical with those governing the manifestations of vital phenomena in the sheep or the dog. We are compelled to believe that the more highly organized brain and nervous system of man constitute an important element in all vital processes, and modify, to some extent, the laws which hold good for the lower animals. We will cite a case in illustration of this remark. A quack announced that he had invented a styptic which would prevent hemorrhage from large arteries, and invited the faculty to witness his experiments. In presence of some physicians, he severed the carotid of a sheep, applied his nostrum, and the animal went on its way rejoicing, much to the amazement of the doctors and the glory of the charlatan. Presently, however, it came to be known that the carotid of a sheep might be cut across without a fatal result, even when no measures were adopted to arrest the flow of blood. This, which we know could, by no possibility, occur in man, is cited to sustain what we remarked above, that experiments performed upon brutes, will never perfectly elucidate the laws which govern man's physical structure. We do not bring forward these suggestions against the volume before us, or for the purpose of casting discredit upon the experiments which were undertaken with the view of discovering the laws of animal life, but for the purpose of warning our readers against falling into the error of supposing that an animal is merely a superior piece of machinery governed by the general laws of mechanics, and driven by the force of chemical affinity, or that man's physical laws are identical with those which apply to the brutes.

We regard the volume before us as being valuable not only for the interesting matter it contains, but for the bearing it must have on the advancement of physiology. Where the chemist leaves off, the physiologist commences, and all will, after a moment's reflection, see how important it is that the starting point of the latter should be well established, in order that should there be error in his calculations or speculations, it may be rectified by recurrence to a basis which is known to be correct.

It is beautiful to see how one science simplifies and elucidates another. The wild vagaries of the humoral pathologists, founded on false physiological doctrines, and ignorance of organic chemistry, were overturned by the solidists, who were equally in error in overlooking the influence of chemical action upon the animal economy; but when the researches of Liebig, Berzelius, and others, were announced to the scientific world, physiologists began anew to construct their theories on a more rational basis, and pathologists began to discover that, as in other cases, truth lay between the extremes contended for by the rival sects. The investigation of physico-chemical phenomena will henceforth constitute a distinct department of scientific research, the foundation of which may be considered as erected by Prof. Matteucci. This research will doubtless be prosecuted with much vigor, and be productive of valuable results.

We pass over that portion of the work devoted to the consideration of imbibition and capillarity, not because this subject is by any means devoid of interest, but in order to devote more space to the consideration of the highly interesting, but not generally understood, phenomena of endosmose and exosmose. We shall not stop to enter into the discussion as to whether the discovery was first made by Dutrochet or not, but proceed to present a brief *expose* of the experiments of Prof. Matteucci, and the practical deductions to be derived therefrom. An endosmometer consists of a glass tube whose lower end is closed by an animal membrane; within this tube is placed an aqueous solution of gum, sugar, &c.; the lower extremity is then plunged into water, when, even contrary to the force of gravity, the water will permeate the membrane so as to elevate the fluid in the tube, while a certain quantity of the solution placed in the tube, will find its way into the reservoir beneath. The first of these results is styled *endosmose*, the latter *exosmose*. As a general rule, endosmose takes place from the rarer to the denser liquid; to this alcohol forms an exception, the water being generally attracted to it. The rapidity with which endosmose takes place, varies generally in proportion to the excess of density of the interior over the exterior liquid. But solutions of the same density when composed of different substances, vary in the degree to which they produce endosmose; thus representing the intensity with which endosmose takes place from water to a solution of gelatin by 3; that when gum is substituted for the gelatin will be 5; with sugar 11; and with albumen 12. There is a singular phenomenon observable when certain acids are placed within the endosmometer. Thus when we use hydrochloric acid of the specific gravity of 1·02, endosmose takes place from the water to the acid; but if the acid have a density of only 1·015, the current sets in the opposite direction. The force of this current, when syrup is used as the interior liquid is estimated as sufficient to raise a column of mercury one hundred and twenty-seven inches, which is a pressure about equal to four and a half atmospheres. Of course various theories have been put forth to explain endosmose and exosmose; all of which our author demolishes, without offering any of his own, contenting himself with stating the conditions necessary to the production of the phenomenon, which conditions he supposes to be—1st, that both fluids should have an affinity for the membrane; and second, that they should readily mix with each other.

It will be at once perceived that this discovery must have a very wide application in the explanation of the phenomena of living beings, and we are tempted to give a summary of these applications, though in so doing we depart from the order observed by the author of the work before us. The experiments of Matteucci were very ingenious, and varied in all conceivable ways. He used the skins of various animals, as well as their stomachs and bladders, turning their different surfaces first to the water and then to the solution contained in the endosmometer, carefully noting the differences in each experiment. These differences are very curious and worthy of notice. With the skin of the torpedo, placed with its external surface towards the interior of the endosmometer, the contained fluid rose thirty millimetres,* while, when the membrane was reversed, the fluid attained a height of only six to eight millimetres. Nearly the same results were obtained with the skins of the frog and eel.

The second class of experiments was performed with the stomach of the lamb, cat, and dog, and the mucous membrane of the gizzard of a fowl. Placing the internal surface of the stomach of the lamb towards the interior of the instrument, the fluid rose 56 millimetres; reversing the membrane, the fluid reached a height of sixty-six to seventy-two millimetres. With the solution of the white of an egg, a contrary effect was produced; with a solution of gum arabic, the elevation was very slight. Using the stomach of the cat with a solution of sugar, the elevation was thirty-eight millimetres; reversing the membrane, the elevation was fourteen millimetres. The same was observed with the stomach of the dog. With the mucous membrane of the stomach of a fowl, the difference of elevation is not much influenced by the position of the membrane; and, contrary to what takes place with other membraees, endosmose always takes place from alcohol to water.

The third class of experiments was performed with the urinary bladder of the ox. Placing the internal surface towards the interior of an endosmometer containing a solution of sugar, the liquid rose to the height of eighty to one hundred

* A millimetre is 0·03937 of an inch.

and thirteen millimetres; reversing the membrane, it rose to sixty-three to seventy-two. With a solution of gum arabic the membrane being in the first mentioned position, the elevation was only from seven to eighteen millimetres, while in the reverse position it was from twenty-two to fifty-two. We give in the author's own words, the conclusions at which his numerous experiments enabled him to arrive.

1st.—The membrane interposed between the two liquids, is very actively concerned according to its nature, in the intensity and direction of the endosmotic current.

2d.—There is, in general, for each membrane, a certain position in which endosmose is most intense; and the cases are very rare in which, with fresh membrane, endosmose takes place equally, whatever be the relative postion of the membrane to the two liquids.

3d.—The direction which is most favorable to endosmose through the skins, is usually from the internal to the external surface, with the exception of the skin of the frog, in which endosmose, in the single case of water and alcohol, is promoted from the external to the internal surface.

4th.—The direction favorable to endosmose through stomachs and urinary bladders varies, with different liquids much more than through skins.

5th.—The phenomenon of endosmose is intimately connected with the physiological condition of the membranes.

6th.—With membranes dried or altered by putrefaction, either we do not observe the usual difference arising from the position of their surfaces, or endosmose no longer takes place.

From a detail of the foregoing experiments on the skins of those animals which live in the water, it will be seen how admirably nature has provided for their preservation and comfort. All are familiar with the peculiar character of the skin of the eel. By referring back to the experiments detailed above, it will be noticed that endosmose from water to gum and albumen, is much weaker when the exterior of the skin is turned towards the water; were it otherwise these animals would become, as it were, water-logged. The same is doubtless true of all aquatic and amphibious animals from whose skins a mucus secretion is discharged. From the experiments performed upon the stomachs of animals, we are at once struck by the differences exhibited by those of the herbiverous and carnivorous classes, and it would be a matter of the highest interest to to pursue the investigation of this subject with the view of ascertaining more exactly the difference in the digestive processes of the two classes of animals though in a process so complicated as digestion, this inquiry must necessarily prove one of much difficulty.

The elementary cell doubtless owes its nutrition to this property of membranes, as do the ovules in the oviducts of mammiferous animals. In this way may be explained the actions of certain remedies in the system. It was noticed by Poisenille, that endosmose took place through animal tissues from the serum of the blood to certain saline solutions. Now the action of purgatives of this class is to produce copious serous evacuations, and without asserting that this effect is produced solely by the endosmotic action, there is every reason to puspose that this effect is produced solely by endosmotic action, pose that this property of the membranes is concerned in the action of purgatives of this nature. On the other hand it has been observed that morphia introduced into a saline solution, checks the current from the serum to the salt, and ultimately reverses it. This serves, in a great degree to explain the action of opium, and may, perhaps illustrate the action of the whole catalogue of astringents. The use of saline remedies in conjunction with opiates, has been suggested as a method of treatment in cholera, and we are bound to bel'sve the suggestion is at least philosophical; how far it is practically valuable we know but little.

In the few experiments performed with the intention of elucidating the phenomena of endosmose, it has been found that when a pair of lungs are partially inflated with oxygen, and introduced into a jar of carbonic acid inverted over water, the lungs become distended so as entirely to fill the vessel; and upon analysis it was found that the gas contained in the lungs consisted of two-thirds oxygen and one-third carbonic acid; that in the jar being composed of one-fourth oxygen and three-fourths carbonic acid. On inflating the lungs to the extent of their capacity with carbonic acid, and immersing them in a jar of oxygen the lungs collapsed, and it was found that the bulk of the carbonic acid which escaped was greater than that of the oxygen which found its way into he pulmonary cells. These experiments would seem to indicate that the endosmotic current sets from carbonic acid to oxygen. There is a difficulty in applying these experiments to the explanation of the phenomena of respiration; for the bulk of carbonic acid exhaled from the lungs never exceeds that of the oxygen introduced, but, according to some authors it is less.

It would be interesting to enquire to what extent many other of the processes of the animal economy are influenced by the endosmotic property of membranes; as, for instance, absorption and exhalation; but having already occupied considerable space with our remarks on endosmose, we will, for the present, take leave of the subject, hoping that some of our readers may be instigated to pursue the subject and furnish us with the fruit of their researches.

Lecture IV is taken up by a consideration of the phenomena and doctrine of absorption and exhalation. It was for a long time supposed that the lacteals and lymphatics were the only vessels concerned in this process; but recent investigations have demonstrated that not only these, but the arteries and veins are actively engaged in it. It was also supposed that the mouths of vessels opened along the track of the intestinal tube for the purpose of absorbing the chyle and carrying it to the thoracic duct; but microscopic anatomy has shown that there is no such thing as an open vessel in the human economy; the arteries terminate in the veins, and the lymphatics form an uninterruptedly continuous network. Absorption, therefore, consists in imbibition and transmission, and all the vessels participate in the function.

The only conditions necessary to it, and an experiment illustrative of those conditions, are thus set down by the author:

*Physical Conditions of Absorption.*—In a word, absorption is always effected under the following conditions:---

1st.—A vessel with organic sides or walls.

2d.—An anterior liquid capable of being imbibed by the tissue composing the walls.

3d.—An internal liquid, also capable of being imbibed by the walls; of intermixing with the exterior liquid; and of circulating in the vessel with more or less rapidity.

Nothing, consequently, can be more physical than a phenomenon thus constituted. I will demonstrate, by an experiment, the truth of this assertion. Here is a long piece of vein taken from a large animal; it is attached at one end to a tube connected with an opening in the lower part of the side of a glass bottle; the other extremity is tied to a small bent glass tube, furnished with a stop cock. I fill the bottle and consequently the vein also, with water. I immerse a portion of the vein in water acidulated with sulphuric or hydro-chloric acid. At first the liquid in the bottle gives no indication of the presence of the acid, but after a certain time, it does. If, instead of waiting some time, and leaving the liquid in repose, I open the stop-cock, I can immediately detect the presence of the acid in the liquid which flows out, but in the bottle it is not yet discoverable. That which happens with a portion of vein, will take place in the same way with the arterial trunk, and with tubes of clay, pasteboard, and wood. If the acidulated solution be contained in the interior of the vein; and if into the liquid of the basin, in which the vein is immersed, we pour some tincture of litmus, the same phenomenon is observed, that is, the acid will pass out through the coats of the vein, with a facility proportionate to the velocity of the current. The conditions of the phenomenon, are always the same; two liquids miscible with each other, and separated by a membrane capable of imbibing them, and the movement of the internal liquid which carries in a given direction, the liquid which has traversed the membrane.

Suppose, for a moment, that the direction of the circulation of the blood was inverse to that which it really is, but without any alteration of the structure and disposition of the blood-vessels; we should then no longer say that the veins absorbed, but, on the contry, that absorption took place by the arteries. Such is the very simple physical phenomenon of that function.

A few words may be necessary as to the changes wrought by the influence of absorption. If we place a colored solution on a filter of bone black, the liquid passes through while the coloring matter is retained. So with membranes; some substances will pass through them, others will not, and it is therefore fair to infer that many of the flnids of the body, are susceptible of modification in this way. It was formerly held that the secreting organs, such as the liver and kidneys, were merely pieces of filtering apparatus. Now although this may appear to be a coarse conception, there may be more truth in it than would appear at first sight, but further researches are needed to elucidate this obscure subject.

Lecture V is devoted to the consideration of digestion. Alimentary substances are divided into three classes. First, the nitrogenized compounds; second, fatty matters, and third, that class including starch, gum, and sugar. Prof. Matteucci regards fibrine, caseine, and albumen as identical in composition. This, though nearly correct, is not absolutely true as can be seen by reference to almost any of the standard works on chemistry. By reference to a foot note by Dr. Pereira it will be further observed that the existence of the compound announced by Mülder as the base of the albumen-fibrine and caseine, and termed by him proteine, is by no means well established. Late researches of Liebig throw much doubt over this theory, as well as the isomerism of the three elements above mentioned. This is a fact well worthy of attention. We are disposed at this time to adopt the opinions of Liebig, notwithstanding the experiment of Denis which is cited by Matteucci as proof positive of the isomerism of these bodies. The experiment was this. Fibrine is converted into albumen by being dissolved in a satuated solution of nitrate of potassa, but this holds true only for the fibrine of the venous blood, that from the arteries undergoing no such change. Venous fibrine when exposed to the action of oxygen lost this property, the same time converting the oxygen into carbonic acid. Now it appears to us that this experiment proves nothing except that the fibrine of the venous blood is characterized by some peculiarties, which the fibrine of the arterial blood does not posess; and that so far from being isomeric with albumen and caseine, it is not constant in its own chemical composition. There is another fallacy in this experiment: there is a probability that there is chemical reaction between the nitrate of potassa and the fibrine, which might account for the phenomenon. What would we think of the man who would tell us that sulphuric acid and sulphate of potash were isomeric, and assign as a reason for his belief that one was converted into the other by being introduced into a solution of nitre. To say the least of this experiment it lacks exactness. We should have been informed whether the dissolved fibrine was found to be identical with that of the venous blood, and whether the solution of the nitrate was in no respects changed in its chemical character during the process.

An illustration of that wonderful class of phenomena termed catalytic, or actions of contact, is afforded by the function which pepsine performs in the the process of digestion, influencing the process without taking any particular part in it; as a boy sets two dogs to fighting without espousing the cause of either. The action of this is analogous to that of diastase in the vegetable world, and platinum sponge upon a mixture of hydrogen and oxygen. The modus operandi of this article, together with other interesting matters pertaining to the process of digestion, will be found in the subjoined extract.

I have shown you, in glasses, an infusion of pepsine to which a few drops of hydrochloric acid have been added. Into one of these small glasses has been put some coagulated albumen; into another some fibrine. The vessels thus prepared have been placed for ten or twelve hours in an atmosphere heated to thirty degrees centig. [eighty-six degrees Fahr.] and the albumen and fibrine have already in a great measure disappeared, there remain only some small fragments which are already transparent on the edges, and which will shortly entirely disappear. If I neutralize the acid and then evaporate the solution, I can easily reproduce the albumen and fibrine, which have not been changed in their nature, but have merely dissolved by contact with the acid infusion of pepsinė. This substance acts, therefore, in the solution of fibrine and albumen, as a body endowed with catlytic properties, and their solution is effected by an action of contact. It is only in the stomach, or by certain glands situated in the mucous membrane of this viscus, that the acid solution of pepsine or the gastric juice is separated. I have tried the effect of placing pieces of the small or large intestines in a very weak solution of hydrochloric acid, the solution never acquired a solvent property—it became gastric juice only by contact with the membranes of the stomach.

The property with which pepsine is endowed requires the, constant presence of free mineral organic acid. If, on the other hand, pepsine be dissolved in an alkaline liquid, its catalytic action becomes modified, as we shall hereafter find.

Lastly, I may remark, that pepsine looses its properties and becomes invaluble when heated beyond 50° centig, [122 Fahr.]

Azotized neutral substances, dissolved in the stomach by the acid liquid, or by the catalytic action of pepsine, pass into the blood mearly by the imbibition of the cappillary blood-vessels of the stomach. Water, and colored alcoholic drinks, introduced into the stomach, are also absorbed; they do not pass beyond this viscus, nor are they to be found in the chyle; yet they reach the blood. Bouchardat and Sandras fed animals with fibrine, colored with either saffron or cocheneal, and yet could never detect a trace of the coloring matter in the chyle. Moreover, animals fed on fibrine, and others which were kept fasting, yielded, when killed, chyle always of the same kind: the contents of the intestines in no way differed, except, that in the animals fed on fibrine, a portion of the latter was found in the stomach incompletely dissolved. We know, also, from the celebrated experiments of Tiedman and Gmelin, that the quantity of fibrine contained in the lymph and chyle, after a fast, is not less than that which is formed there after digestion. The results are the same when coagulated albumen, gluten, aud caseous matter, are employed instead of fibrine. The digestion of these azotised neutral substances is, therefore, a mere solution, effected by an action of contact, and an absorption of these taking place chiefly in the stomach. Thus then, nothing is more physical than this part of digestion.

The mastication of aliments impregnated with a slightly alkaline and warm liquid, is an entirely physical operation similar to that which we practice in our laboratories, in order to effect the division of a body, and thereby to promote its solution.

The gastric juice which the stomach secrctes, especially at the moment of digestion, is an infusion of pepsine in acidulated water; and if we cause it to act on coagulated albumen, on fibrine or casein, the solution of these substances can effected as well in a properly warmed receiver as in the stomach.

The movement of the walls of the stomach promotes the action of the infusion of pepsine upon the sabstances to be dissolved, just as all agitation aids the reaction of two dissolved bodies, or the solution of a solid in a liquid.

This movement of the walls of the stomach, is also of assistance in another way, by incessantly removing the points of contact between them an the matter which they contain, the abssorption of the liquid portion of this substance is effected more readily. The influence which the division of the eighth pair of nerves has in disturbing digestion, is ascribable, in part, to the cessation of these movements, which are dependent on the action of the nerves. Moreover, their section produces a great disturbance in other functions indispenable to the integrity of the animal economy.

Recent physiological and anatomical researches, would seem to prove that the germ of all animal tissues is the cell, and it is an interesting investigation to trace the origin of it. Our author's theory of the matter amounts to the following. In the chyle and lymphe are suspended a vast number of minute bodies, from one to two thousandths of a line in diameter, formed of a fatty substance and enveloped in a membrane: several of these unite by some process and form a globule. For a theory of the nourishment of these cells we must refer back to what has been said of endosmose.

In respiration the blood is changed in the lungs from venous to arterial by the absorption of oxygen and evolution of carbonic acid. It was for sometime supposed, and for aught we know to the contrary is still believed, that the lungs were the sole agents concerned in the manufacture of carbonic acid, and that oxygen was introduced into the venous blood only through the medium of the lungs. But it is now known that oxygen may be introducd into the circulation through the medium of the skin. Sorg immersed his arm in oxygen and found at the end of four hours two thirds of it had disappeared. Gasses injected into the pleura disappear, and the same is doubtless true of all the other cavities of the body. Now this being the case, and the veins being absorbents, we may reasonably conclude that carbonic acid is formed in those vessels and carried to the lungs to be eliminated. Fishes will live with their heads out of water provided their bodies are still submerged. Frogs and snakes can exist without lungs, but perish if their skins are varnished. Here is a function of the skin not usually taken note of, but one that is worthy of the attention of the practitioner. It has been found that the gasses found in the blood are carbonic acid, oxygen and nitrogen. In arterial blood the volume of these is greater than in the venous blood: in the former the oxygen is double that in the later, while the venous blood contains a greater proportion of carbonic acid and nitrogen than does the arterial. So far as our knowledge of the phenomena of respiration at present extends, they may be looked upon as purely physico-chemical phenomena. We would gladly dwell awhile longer on this part of the subject, as there are many very interesting topics connected with the theory of animal heat, but there are other matters in the volume which demand a notice.

In lecture VIII we have an account of phosphorescence as exibited in living beings. For this purpose a number of experiments were tried upon the gloworm. The phosphorescent nature of this animal seems to some extent independent of the vital force, as the phenomena does not cease with life. Carbonic acid was found to destroy this property, in a few moments was it again restored by the action of atmospheric air or pure oxygen. When the insects were confined in a limited portion of atmospheric air, the phosphorence after a time disappeared, and it was found upon examination that the oxygen had disappeared and been replaced by an equal volume of carbonic acid. In chlorine the light was soon extinguished, nor could it be restored by the introduction of oxygen. Oxygen sustained the phosphorescence until carbonic acid was evolved equal in bulk to one third of the oxygen originally introduced.

We come next upon the most interesting if not the most important portion of the volume, that which treats of electricity in connection with the phenomena of animal life. In considering this subject there is at the outset a great error to be avoided. Too many are in the habit of regarding electricity as the cause of the various chemical actions which are going on in the body. This is just the reverse of what is true. Electricity is doubtless the effect, not the cause. As before observed we think it a course conception to liken the animal to an electric or galvanic apparatus. In his researches on this subject our author used what he terms the *galvanoscopic* frog; that is the leg of a frog with the crural nerves attached to it. This is introduced into a glass tube, and thus becomes insolated. If we cut into the muscle of an animal and touch with two differant filaments of the nerve, a potion of the muscle on each side of the wound, the muscles of the frogs leg manifests such a contraction as would be produced by bringing it in contact with the electrical apperatus. If instead of the frogs leg, we use in this experiment a delicate galvanometer the same effect will be produced. The evidence of the muscular current is much more clearly demonstrated by the following experiment: take five or six frogs cut them in half, disarticulate the thighs from the legs, and divide the former transversely; take the lower halves of the thighs, and arrange them on a varnished tray having the cup shaped depressions in it. The thighs are arranged in the following manner: the first has its external surface contained in one of the cups of the tray; the next one has its external surface in contact with the internal surface of the first, and so on alternately, the last one having its *internal* surface in one of the cup shaped depressions of the tray. Filling these cups with a saline solution, and bringing the galvanometer in contact with the pile, an electic current is found to travel from the internal to the external surface of the muscles. Very feeble currents have also been observed in other tissues, such as the adipose, glandular &c. It has been found that this current is totally independent of nervous influence, for we presume that no one believes that the nerves exercise any control after death, nor indeed is the experiment in any way varied by depriving the muscles of the nerves which supply them. If in the experiment living frogs are substituted for dead ones, the same result is produced with a much better effect. In an animal badly nourished the electric current becomes weaker; if the muscles have been sobjected to an inflammatory process, the current is augmented. Poisoning the animal produces no effect, but submitting it to the action of sulphuretted hydrogen almost entirely destroys the current. What is the cause of these electrical phenomena? Liebig supposes they are owing to the action between a free acid in the muscles and the alkaline constituents of the blood. This theory our professor thinks untenable, and explains it. by supposiug that it is owing to the contact of the oxygen of the blood with the muscular tissue which becomes transformed in the process.

By experiment it was found that if the spinal marrow was not cut across and a current was passed along the crural nerves, spasmodic action was excited in the muscles of the neck and head, whereas when the integrity of the spinal marrow was destroyed, no such affect was produced. This goes far to illustrate the doctrine of the reflex function of the nervous system, and in this point of view is very valuable. But perhaps the most interesting phenomena, and certainly the most important in a therapeutic point of view, are those exhibited by what are called the direct and inverse current. By the direct current is meant one transmitted in the direction of the nervous ramifications; that is, from the centre to the periphery. By the inverse current is designated that passed from the periphery to the centre. It has been observed that after a time the direct current diminishes to a great extent the excitability of a nerve, while the inverse current has a contrary effect. The therapeutic indications are obvious. If we wish to bring electricity to bear in tetanus, hydrophobia and other diseases accompanied by high nervous excitability, we use the direct current; on the other hand, in paralysis, we would take care to direct the current in the opposite direction. Ignorance of this action of the electric fluid, has doubtless been to a great extent the cause of the failure of this agent in the treatment of disease, for we can at once see that a misapplication of the remedy would aggravate the disease. Are we to infer from this that the nervous force is identical with electricity, and that nervous diseases result from a disturbance of the electric current? Here is what our professor says on this point.

*No electric current in the nerves.*—It was important to search for the presence of an electric current in the nerves of a living animal. I shall refrain from noticing here all the experiments which have been undertaken with this object in view, and which have terminated in the announcement, at one time that this current did exist, at another that it did not exist. The most conscientious and best established conclusion is this:—*In the present state of science, and with the means of experimenting which we at present possess, no sign of the electric current is found in the nerves of living animals.*

Some persons have asserted, that a steel needle introduced into the muscles perpendicularly to the direction of their fibres became magnetic, especially at the moment when the mucles contract. From this it has been concluded, that there existed an electric current in the nerves, and that the circuit was established as in a spiral or an electro-dynamic cylinder.

I have repeated these experiments, by introducing steel or iron needles into the muscles of living animals, and in all directions relative to their fibres. In order to convince myself of the magnetization of the needles, thus plunged into the muscles, I made use of those of a very good astatic system, and even those of the sideroscope of Lebaillif. I never obtained an affirmative result. I placed the recently prepared thigh and leg of a frog, in the interior of a spiral of varnished copper wire, the extremities of which were connected with those of second and smaller one, in which there was a soft iron wire. I afterwards irritated the nerve of a frog, observing at the same time, if an induced current traversed the spiral, and magnetized the iron wire. All my researches were fruitless.

I likewise tried the effect of introducing into an exposed nerve of a living animal, the conductors of a very delicate galvanometer by two points as far apart as possible. I operated upon animals under the influence of certain narcotic poisons, and I excited strong muscular contractions in them, at the moment when I placed the two wires of the galvanometer in the nerve; but I must confess that, whenever the experiment was well made, I never obtained evident and constant traces of the electric current.

At the school of Alfort I made in conjunction with Longet, an experiment of this kind upon a horse. We employed a very delicate galvanometer; the nerve was exposed for a considerable extent of its course, and I could traverse it with the platinum extremities of the galvanometer, by passing from a distance of 2 or 3 centimeters to that of 15 or 20 (from ·78 of an inch to ·7-87 inches). We never obtained distinct signs of the derived current, and in a constant direct- even when the muscles of the animal were violently contracted.

Lastly, I may add that, from what we know of the properties of electricity, and of the laws of its propagation, it is impossible to conceive the existence of a current circulating in the nerves. In order that an electrical current should pass from one extremity to the other, it would be necessary to compare the nerve to a metallic wire varnished or otherwise insulated, an assumption which is not in accordance with fact. An electric current which, subjected to the will, would set out from the brain to reach the muscles, by traversing the nerves.

could not be stopped in its course by the ligature of the nerves; whereas, we well know, that the propagation of the nervous force is prevented by that proceeding. Lastly, its circulation in the nerves requires that the nervous system should form a closed circuit, but the labors of anatomists are very far from having proved such an arrangement, especially in the ultimate ramifications in the muscles, where it would be especially necessary.

The remaining hundred pages of the volume are mainly taken up with the consideration of the voice, the ear, the eye, the mechanism of the muscles, and the circulation. These matters although of much interest we have not time to mention more in detail, contenting ourselves with recommending this valuable little volume to all who wish to enter on the higher walks of the profession, and to all who desire to become what a physician should become, an adept in every science that may be available in the relief of suffering.

*Indianopolis*, *April*, 1849. E. G. M.

